# Discordance between perceived risk and actual risky sexual behaviors among undergraduate university students in mainland China: a cross-sectional study

**DOI:** 10.1186/s12889-022-13132-w

**Published:** 2022-04-12

**Authors:** Yusi Liu, Guochen Fu, Yifan Chen, Lei Wu, Mingliang Pan, Yuli Yang, Zhuo Chen, Yu Cao, Yong Li, Hao Wang, Bixiang Wang, Chengcheng Lv, Ruyi Du, Yanting Xiong, Wei Liu, Nuo Xu, Xiaobao Xia, Qianqian Li, Fang Ruan, Junfang Wang

**Affiliations:** 1 Department of Preventive Medicine, School of Basic Medical Sciences, Hubei University of Science and Technology, No.88 Xianning Avenue, Xianning City, Hubei Province 437100 China; 2 National Demonstration Center for Experimental General Medicine Education of Hubei University of Science and Technology, Xianning City, China

**Keywords:** Sexual risk, Self-perceived risk, Risk discordance, Undergraduates, China

## Abstract

**Background:**

HIV prevention, diagnosis, treatment and care services might be hampered by inaccurate risk assessment. This study aimed to investigate the extent of and factors associated with the discordance between perceived risk and actual risky sexual behaviors among undergraduates in mainland China, guided by the Anderson's behavioral model.

**Methods:**

This study involved a secondary analysis of cross-sectional data collected during the fall semester of 2018–2019 academic year. The present analysis was restricted to 8808 undergraduates with low risk perception. Those who had low perceived risk but actually engaged in risky sexual behaviors were categorized as risk discordance (RD). Univariate and multivariate Logistic regression analyses were conducted to identify factors associated with RD.

**Results:**

Overall, the discordance rate between perceived and actual risk was 8.5% (95% CI: 7.9%-9.1%). Multivariate Logistic regression analysis indicated that non-heterosexual women (AOR = 0.41, 95% CI:0.27–0.60), heterosexual men (AOR = 0.45, 95% CI:0.33–0.61) and women (AOR = 0.26, 95% CI:0.19–0.35) were less likely to exhibit RD, when compared with non- heterosexual men. Furthermore, non-freshmen (AOR = 1.57, 95% CI:1.30–1.90), early initiators of sexual intercourse (AOR = 5.82, 95% CI:4.10–8.26), and those who had lower levels of HIV knowledge (AOR = 1.28, 95% CI:1.08–1.51), displayed higher levels of stigma against PLHIV (AOR = 1.50, 95% CI:1.26–1.77) and had ever been tested for HIV (AOR = 1.36, 95% CI:1.04–1.77) were more prone to reporting RD. Those with more enabling resources [i.e., displaying high levels of condom use self-efficacy (AOR = 0.70, 95% CI:0.59–0.84) and being knowledge of local testing center (AOR = 0.71, 95% CI:0.60–0.83)] were less likely to report RD. However, spending more than 2000 Yuan a month on basic needs (AOR = 2.55, 95% CI:2.07–3.14), residing in urban areas (AOR = 1.35, 95% CI:1.15–1.59) and being knowledgeable of the national AIDS policy (AOR = 1.40,95% CI:1.18–1.66) increased the chance of exhibiting RD.

**Conclusions:**

Comprehensive interventions, including targeting students with high-risk characteristics, improving the acceptability of PrEP and PEP, conducting health education, enhancing self-efficacy for using condoms and making opt-out HIV testing routine in college campus, should be taken to reduce the discordance between perceived and actual HIV risk and finally to reach the goal of Zero AIDS.

## Background

Risk discordance (RD) is commonly used to describe inconsistencies between self-perceived and actual risk of HIV infection. Those who held the inaccurate perception of being at low risk for HIV infection are of particular interest [1–10], because they might fail to adopt HIV risk-reduction approaches, including non-use, incorrect or inconsistent use of condoms and Pre-exposure prophylaxis (PrEP), refusing routine HIV testing, missing opportunities for early diagnosis and treatment of HIV infection, and thus contributing to the ongoing transmission.

RD among undergraduates in mainland China has become the focus of recent research. Frequently engaging in high-risk sexual behaviors and their increasing vulnerability to HIV infection makes this focus understandable. Previous studies related to RD [1–10] were largely conducted in Western countries and mainly focused men who have sex with men (MSM). Furthermore, they only elucidated the effects of individual demographic factors, AIDS-related knowledge, attitude, belief and practice (KABP), but ignored the effects of peer, familial, and social networks and community resources on RD. However, no prior studies have been conducted to evaluate the prevalence of discordance between self-perceived HIV risk and risky behaviors among undergraduates in mainland China. Thus, there is a need for measuring and explaining RD, so that proper solutions can be devised to reduce RD, heighten PrEP acceptability, improve testing uptake, reduce the burden of undiagnosed HIV infections, and finally achieve the goal of zero AIDS. Therefore, drawing on a large, geographically diverse sample of college students from across China, this study aims to assess the extent of and factors associated with the discordance between perceived HIV risk and actual risky sexual behaviors among undergraduates in mainland China.

### Conceptual framework

The Anderson's Behavioural Model [11] has been adapted to elucidate the factors contributing to risk discordance. According to this model, the decision to undergo HIV testing is affected by three sets of factors: predisposing, enabling and need characteristics [12]. Predisposing characteristics include sociodemographics {e.g., gender [12], sexual orientation [12], major [12], grade/age [12, 13], race [2], occupation [8], age at first sex [1, 8, 14]} and AIDS-related knowledge-attitude-belief -practice (KABP) such as knowledge of HIV transmission [1, 12], stigma against people living with HIV (PLHIV) [12] and prior testing [1]. Enabling characteristics refer to peer, familial, and social networks and community resources such as condom use self-efficacy [15], family income [12], geographic region [1, 14], urban or rural area of residence [12, 14], availability of local testing center [12, 14], and political commitment to HIV/AIDS control [14, 16]. Need characteristics include indicators of subjectively (i.e., self-perceived HIV risk) and objectively-assessed risk of HIV acquisition (e.g., risky sexual behaviors). The model also assumes that predisposing and enabling variables exert effects on the utilization of HIV testing service directly as well as indirectly through their influence on need characteristics [11].

### Objectives

**Fig. 1:**
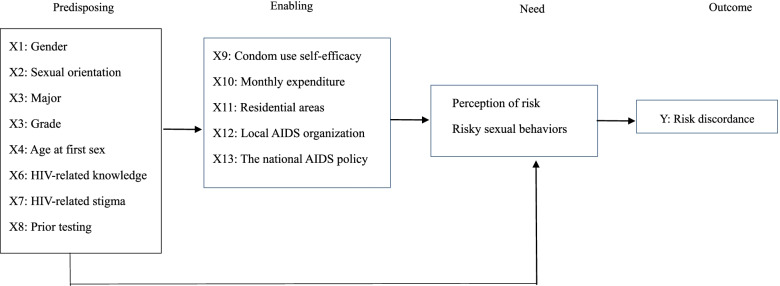
Individual determinants of risk discordance adapted from the Andersen's model of health service utilization

Therefore, guided by the Anderson's behavioral model and based on the findings from previous studies, thirteen independent variables hypothesized to influence risk discordance (RD, defined as the mismatch between self-perceived and actual risk of HIV infection) were first organized into predisposing characteristics and enabling resources, as indicated in Fig. 1. Logistic regression models were then chosen to estimate their respective effects on RD so as to design interventions to reduce RD and finally to reach the goal of Zero AIDS. RD is hypothesized to be mainly determined by early initiation of sexual intercourse. It is also hypothesized that non-heterosexual men and those with less enabling resources would be more likely to report RD. The role of other predisposing factors is examined in a more exploratory fashion.

## Methods

### Study design and settings

This study involved a secondary analysis of cross-sectional data collected during the fall semester of 2018–2019 academic year. The original survey was conducted by the Hubei University of Science and Technology (HUSC). Briefly, a total of 21,184 participants currently enrolled as a full-time undergraduate student at HUSC were first organized to complete a web-based questionnaire with questions on demographic characteristics, knowledge, attitude and behaviors towards HIV/AIDS, due to their convenience and better cooperation. Furthermore, a series of measures such as giving them course credits and rewarding them with a certain amount of money were adopted to increase their participation rates. In addition, Wechat and Sina Weibo, as the largest social media platforms, were also used to distribute questionnaires to improve sample representativeness. A detailed description of the study design and settings was demonstrated in our previous publications [12, 17, 18].

### Participants

The original survey involved a total of 12,750 participants distributed across the Chinese mainland (except for Tibet). However, the present study was restricted to 8808 undergraduates with low risk perception who must meet the following inclusion criteria: (a) aged 18–25 years; (b) full-time undergraduates; (c) currently registered at one university in mainland China; (d) answered the online questionnaire no later than January 9, 2019; (e) perceived themselves to be at no or low risk for HIV infection.

### Sample

**Fig. 2:**
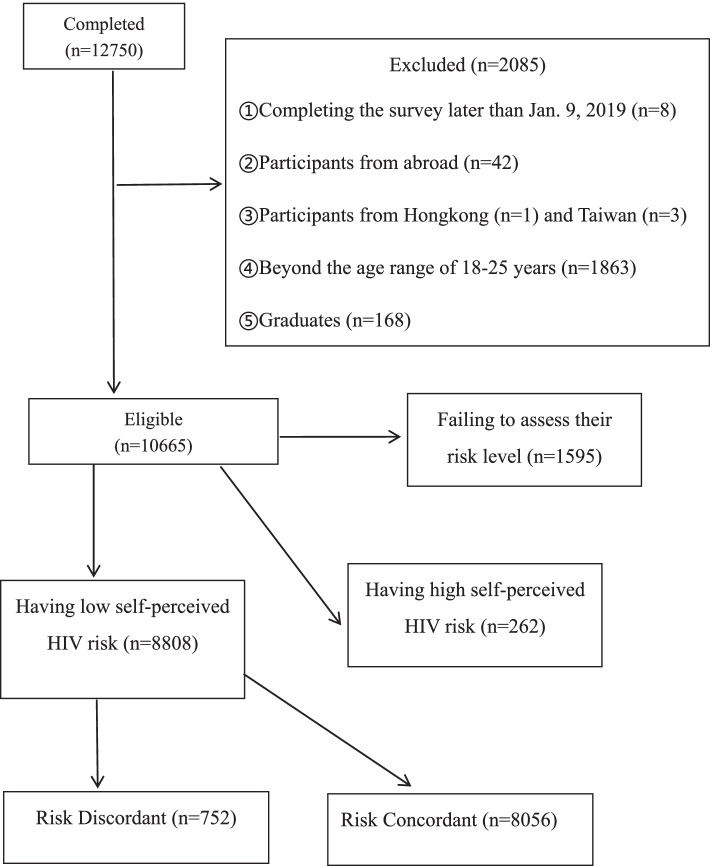
Flow chart showing study sample selection

The details of the study sample selection were shown in Fig. 2. As mentioned above, the data were extracted from a total of 12,750 completed surveys. 2085 subjects were first excluded because of completing the survey later than January 9, 2019 (*n* = 8), coming from abroad (*n* = 42), Hongkong (*n* = 1) or Taiwan (*n* = 3), falling beyond the age range of 18–25 years (*n* = 1863) and being a graduate student (*n* = 168). Then respondents who failed to assess their risk level (*n* = 1595) and perceived themselves to be at moderate (*n* = 87) or high (*n* = 175) risk of acquiring HIV infection [i.e., having high self- perceived HIV risk (*n* = 262)] were excluded. Finally, 8808 subjects who self-identified as having no (5526) or mild (3282) risk of HIV infection [i.e., having low self-perceived HIV risk (*n* = 8808)] were selected as study sample.

### Instruments

Based on the theoretical framework of the Anderson's behavioral model, a self- developed and validated structured questionnaire was used to collect predisposing, enabling and need factors, as indicated in Fig. 1.

### Predisposing

Eight predisposing characteristics included gender, sexual orientation, major, grade, age at first sex, HIV-related knowledge and stigma, and prior testing. Given the fact that male students who have sex with men (MSM) are disproportionately infected or at risk for HIV, a combined analysis of gender and sexual orientation was undertake and was then split into four different groups (non-heterosexual men & women, and heterosexual men & women), and "non-heterosexual men" was chosen as the reference group when performing multivariate Logistic regression analyses. Grade was crudely used to measure their age and categorized into two groups (0 = Freshmen, 1 = Non-freshmen), since Chinese university students in the same grade are almost of the same age. In this study, the age at first sex was established as an independent variable, rather than the dependent variable (i.e., derive the risky behaviour variable) because respondents had their first sexual experiences at different stages of their lifecycles [14]. Participants who had their first sex younger before the age of 14 years were categorized as early initiators, while those who had no sexual experience or had first sex at 15 years or older age were classified as late initiators.

Consistent with our previous study [19], HIV-related knowledge was measured by the 12-item scale of Yes/No/I do not know questions (Cronbach's α = 0.73), while stigma and discrimination against people living with HIV (PLHIV) was based on the Chinese version of Zelaya's 24-item scale of Yes/No/It depends on the situation statements (Cronbach's α = 0.92). Both knowledge and stigma scores were not normally distributed and therefore were categorized into two groups according to their respective median value. In this study, subjects were classified into two groups (having ever been tested or having never been tested) according to their responses to the Yes/No question "have you been tested for HIV?".

### Enabling

Five enabling characteristics in this study included condom use self-efficacy, monthly expenditure, residential areas, knowledge of local AIDS organization and awareness of the national AIDS policy. Consistent with our previous study [17], condom use self-efficacy was measured by asking the participants whether they felt confident they can negotiate use of condoms when faced with eight different sexual situations (e.g., “Can you use a condom even if sexual partner does not want to?”). Those who answered "yes" to all these questions were categorized as having high self-efficacy, while their counterparts-who answered "No" or "Not sure" to any of the above questions-were classified as having low self-efficacy (Cronbach's α = 0.91).

Since 2003, the central government has introduced and implemented the "Four Frees and One Care" policy, including providing free anti-retroviral medicine to AIDS patients with financial difficulties, free and anonymous voluntary counselling and testing, free drugs in the prevention of mother-to-child transmission of HIV infection, and free schooling of orphans, and care and economic assistance to the households of people living with HIV [16]. Awareness of the national AIDS policy was measured by asking whether the participants had heard of the "Four Frees and One Care" policy and three possible responses (Yes/No/Not sure) were provided. In the final analysis, those who answered "Yes" were classified as "awareness", while those who answered "No" or "Not sure" were classified as "unawareness" of the national AIDS policy.

### Need

In this study, perception of HIV risk and risky sexual behaviors were respectively used to measure the undergraduate's subjective and objective need for HIV testing. Undergraduates were first asked to estimate their risk of acquiring HIV and five choices were provided: 1 = no risk at all, 2 = I don't know, 3 = low risk, 4 = medium risk, and 5 = high risk. Meanwhile, three types of risky sexual behaviors, including having more than one sexual partner in the past six months, having sex with non-steady sexual partners (e.g., commercial sex, casual sex or one night stand) or failing to condom use consistently in every act of sexual intercourse, were used to reflect actual risk of becoming infected with HIV [17–19].

Consistent with Seekaew et al.'s study [1], risk discordance (RD) was examined by comparing responses to the self-perceived HIV risk question against actual HIV infection risk categories. More specifically, participants who perceived themselves not to be at risk or to be at low risk of HIV infection (i.e., having low risk perception), but actually engaged in at least one of three types of risky sexual behaviors were classified as RD, while their counterparts (i.e., having low risk perception and not engaging in risky sexual behaviors) were classified as risk concordance (RC).

### Data analysis

The procedure to identify factors significantly associated with RD was conducted in the following four steps. First, descriptive statistics were used to characterize the study sample. Second, differences between the groups (i.e., included vs. excluded and RD vs. RC subgroups) were examined using Pearson's Chi-square tests. Thirdly, a multicollinearity analysis was performed to identify the relationship between the independent variables and eliminate redundant variables before multivariate Logistic regression analysis. The term multicollinearity refers to the phenomenon in which two or more variables are highly interrelated in a multiple regression model and high multicollinearity leads to misleading results [20, 21]. A common way to control multicollinearity is to eliminate redundant independent variables from the model. Multicollinearity was examined by calculating the correlation matrix of independent variables and by evaluating statistics parameters such as tolerance and variance inflation factor (VIF, defined as the reciprocal of tolerance) [21, 22]. A common rule of thumb is that the correlation coefficients between the variables are below 0.6 and tolerance values are greater than 0.10 (or VIF values are lower than 10) [22]. Finally, univariate and multivariate Logistic regression analysis were conducted to identify factors associated with RD. The factors that were statistically significant in the univariate analysis were further subjected to a backward stepwise multivariable logistic regression analysis. Only variables with P values less than 0.05 were retained in the final model. The adjusted odds ratios (AOR) and the corresponding 95% confidence intervals (CI) were also presented. All statistical analysis was performed using IBM SPSS Statistics 28.0.

## Results

### Descriptive analysis

Descriptive statistics of the study sample were presented in Table 1. Out of the 8808 students with low risk perception, 752 (8.5%, 95% CI:7.9%-9.1%) reported to engage in at least one of the above-mentioned three types of risky sexual behaviors [including inconsistent use of condoms (*n* = 637), having sex with non-steady partners (*n* = 146), or having more than one partner in the past six months (*n* = 123)], and were thus classified as risk discordant (RD), while their counterparts (i.e., having low self-perceived risk of acquiring HIV and not engaging in risky sexual behaviors) were classified as risk concordant (RC) (Table 1).

**Table 1:** Predisposing and enabling characteristics of the 8808 undergraduates with low risk perception in mainland China

Variable	Inclusion (*n*=8808)		Exclusion (*n*=1595)		χ2	P	Discordant (*n*=752)		Concordant (*n*=8056)		χ2	P
	n	%	n	%			n	%	n			
X1: Gender												
0=Female	5027	57.1	986	61.8	12.47	<0.001	317	58.5	4710	42.2	74.69	<0.001
1=Male	3781	42.9	609	38.2			435	41.5	3346	57.8		
X2: Sexual orientation												
0=Heterosexuals	7874	89.4	1352	84.8	28.87	<0.001	615	81.8	7259	90.1	50.28	<0.001
1=Non-Heterosexuals	934	10.6	243	15.2			137	18.2	797	9.9		
X3: Major												
0=Non-Medical	6146	69.8	1150	72.1	3.48	0.062	525	69.8	5621	69.8	0.00	0.982
1=Medical	2662	30.2	445	27.9			227	30.2	2435	30.2		
X4: Grade												
0=Freshmen	2447	27.8	506	31.7	10.33	0.001	159	21.1	2288	28.4	18.06	<0.001
1=Non-freshmen	6361	72.2	1089	68.3			593	78.9	5768	71.6		
X5: Age at first sex (Years)												
0=14 (Late)	8639	98.1	1582	99.2	9.57	0.002	682	90.7	7957	98.8	238.59	<0.001
1=14 (Early)	169	1.9	13	0.8			70	9.3	99	1.2		
X6: HIV-related knowledge (Range: 0-12)												
0=High (≥8)	5654	64.2	833	52.2	82.38	<0.001	408	54.3	5246	65.1	35.32	<0.001
1=Low (8)	3154	35.8	762	47.8			344	45.7	2810	34.9		
X7: HIV-related stigma (Range: 0-24)												
0=Low (≥18)	4144	47.0	723	45.3	1.60	0.206	245	32.6	3899	48.4	69.09	<0.001
1=High (18)	4664	53.0	872	54.7			507	67.4	4157	51.6		
X8: Testing history												
0=Never	8204	93.1	1445	90.6	13.03	<0.001	675	89.8	7529	93.5	14.72	<0.001
1=Ever	604	6.9	150	9.4			77	10.2	527	6.5		
X9: Condom use self-efficacy (Range: 0-8)												
0=Low (0-7)	5674	64.4	1249	78.3	117.01	<0.001	518	68.9	5156	64.0	7.15	0.008
1=High (=8)	3134	35.6	346	21.7			234	31.1	2900	36.0		
X10: Monthly expenditure												
0=Low (2000 Yuan)	8004	90.9	1501	94.1	17.92	<0.001	601	79.9	7403	91.9	118.89	<0.001
1=High (≥2000 Yuan)	804	9.1	94	5.9			151	20.1	653	8.1		
X11: Residential areas												
0=Rural	5890	66.9	1135	71.2	11.33	0.001	429	57.0	5461	67.8	35.81	<0.001
1=Urban	2918	33.1	460	28.8			323	43.0	2595	32.2		
X12: Knowledge of local AIDS organization												
0=No	4293	48.7	820	51.4	3.86	0.050	414	55.1	3879	48.2	13.12	<0.001
1=Yes	4515	51.3	775	48.6			338	44.9	4177	51.8		
X13: Awareness of the national AIDS policy												
0=awareness	5645	64.1	1214	76.1	86.92	<0.001	428	56.9	5217	64.8	18.39	<0.001
1=awareness	3163	35.9	381	23.9			324	43.1	2839	35.2		

The predisposing characteristics revealed that 57.1% of the students were female and 10.6% identified themselves as non-heterosexuals. Nearly one-third of them were medical students (30.2%) and freshmen (27.8%). A tiny proportion (1.9%) had their first sex younger than 14 years old. The findings also demonstrated low levels of knowledge about HIV transmission (64.2%), high levels of stigmatizing attitude towards HIV-infected patients (53.0%), and the extremely low rate of testing (6.9%).

In terms of enabling characteristics, 35.6% were wholly confident that they could negotiate condom use with their sexual partners under eight different situations, 9.1% reported their monthly expenditure was more than 2000 Yuan RMB, 66.9% resided in rural areas. Furthermore, only 51.3% knew about local AIDS service organization and even fewer (35.9%) were knowledgable about the national AIDS policy.

### Pearson’s Chi-square tests

A comparison between 1595 subjects excluded from and those included in the present analysis was shown in Table 1. As shown in Table 1, there were statistically significant differences between these two samples across all characteristics except for major and HIV-related stigma. Similarly, except for major, all other twelve independent variables had statistically significant associations with risk discordance, as indicated in the last column in Table 1.

### Multicollinearity diagnosis

The zero-order Pearson correlations matrix for analyzing the relationships between independent variables were presented in Table 2. The maximum correlation coefficient (0.25) between HIV-related knowledge (X6) and stigma (X7) was found to be statistically significant (*P* < 0.001), but fell below the criteria (0.60) for diagnosing the collinearity. Additionally, tolerance (ranging between 0.88 and 0.99) and VIF values (ranging from 1.01 to 1.13) (See Table 3) were all within acceptable limits (i.e., tolerance values were all greater than 0.10 and VIF values were lower than 10), suggesting again that there were no signs of multicollinearity among independent variables. Therefore, all of them can be included in the same multivariate Logistic regression model as individual dummy predictor variable.

**Table 2:** The matrix of Pearson correlation coefficients of factors associated with risk discordance

	X1	X2	X3	X4	X5	X6	X7	X8	X9	X10	X11	X12	X13
X1: gender	-												
X2: Sexual orientation	-0.06^***^	-											
X3: Major	-0.02^*^	0.02^*^	-										
X4: Grade	-0.08^***^	0.02^*^	0.05^***^	-									
X5: Age at first sex	0.08^***^	0.05^***^	0.04^***^	0.00	-								
X6: HIV-related knowledge	-0.02	0.03^**^	-0.12^***^	-0.02^*^	0.08^***^	-							
X7: HIV-related stigma	0.04^***^	0.02^*^	-0.07^***^	0.06^***^	0.11^***^	0.25^***^	-						
X8: Testing history	0.07^***^	0.05^***^	0.05^***^	0.03^**^	0.01	0.02^*^	0.02^*^	-					
X9: Self-efficacy of condom use	-0.02	0.03^**^	0.04^***^	0.06^***^	0.07^***^	-0.14^***^	-0.10^***^	0.02^*^	-				
X10: Monthly expenditure	0.03^**^	0.03^**^	-0.04^***^	0.02^*^	0.00	0.01	0.04^***^	0.01	0.05^***^	-			
X11: Residential areas	0.04^***^	0.05^***^	0.01	-0.01	0.03^**^	-0.01	0.01	0.02^*^	0.07^***^	0.18^***^	-		
X12: Knowledge of local AIDS organization	0.02	-0.02^*^	0.15^***^	0.02^*^	0.04^***^	-0.09^***^	-0.06^***^	0.06^***^	0.11^***^	-0.02^*^	0.02^*^	-	
X11: Awareness of the national AIDS policy	0.06^***^	0.02^*^	0.16^***^	0.00	0.13^***^	-0.10^***^	-0.08^***^	0.09^***^	0.19^***^	0.01	0.05^***^	0.22^***^	-

^*^P 0.05, ^**^P 0.01, and ^***^P≤ 0.001

**Table 3:** Multiple linearity diagnosis of factors associated with risk discordance

	Tolerance	VIF
X1: gender	0.97	1.03
X2: Sexual orientation	0.99	1.01
X3: Major	0.95	1.06
X4: Grade	0.98	1.02
X5: Age at first sex	0.95	1.05
X6: HIV-related knowledge	0.90	1.11
X7: HIV-related stigma	0.91	1.10
X8: Testing history	0.98	1.02
X9: Self-efficacy of condom use	0.93	1.08
X10: Monthly expenditure	0.96	1.04
X11: Residential areas	0.96	1.04
X12: Knowledge of local AIDS organization	0.93	1.08
X11: Awareness of the national AIDS policy	0.88	1.13

### Univariate and multivariate logistic regression analysis

The results from the univariate and multivariable logistic regression analysis of factors associated with risk discordance were presented in Table 4. Consistent with the above-described results, only major was found to be insignificantly associated with RD. After adjusting for potential confounding variables, significant associations between RD and the above twelve independent variables still existed. Among all the significant predictors, the adjusted odds ratio (AOR) was the highest for age at first sex. Undergraduates who had their first sex before 14 years old (i.e., early initiators) were nearly six times more likely (AOR = 5.82, 95% CI:4.10–8.26) to report RD than those who initiated sexual intercourse at later ages. Compared with non-heterosexual men, non-heterosexual women (AOR = 0.41, 95% CI:0.27–0.60), heterosexual men (AOR = 0.45, 95% CI:0.33–0.61) and women (AOR = 0.26, 95% CI:0.19–0.35) were less likely to exhibit RD. Furthermore, non-freshmen (AOR = 1.57, 95% CI:1.30–1.90) and undergraduates who had lower levels of HIV knowledge (AOR = 1.28, 95% CI:1.08–1.51), displayed higher levels of stigma against PLHIV (AOR = 1.50, 95% CI:1.26- 1.77) and had ever been tested for HIV (AOR = 1.36, 95% CI:1.04–1.77) were more prone to reporting RD.

**Table 4:** Univariable and multivariable Logistic regression analysis of factors associated with risk discordance

**Variables**	Univariable			Multivariable		
	COR	95% CI	*p*-value	AOR	95% CI	*p*-value
X1 and X2: Gender and Sexual orientation (Ref: Non-heterosexual men)						
Non-heterosexual women	0.35	0.24–0.51	< 0.001	0.41	0.27–0.60	< 0.001
Heterosexual men	0.37	0.28–0.49	< 0.001	0.45	0.33–0.61	< 0.001
Heterosexual women	0.20	0.15–0.26	< 0.001	0.26	0.19–0.35	< 0.001
X3: Major (0 = Non-Medical, 1 = Medical)						
X4: Grade (0 = Freshmen, 1 = Non-freshmen)	1.48	1.23–1.77	< 0.001	1.57	1.30–1.90	< 0.001
X5: Age at first sex (Years) (0 = 14,1 = 14)	8.25	6.02–11.31	< 0.001	5.82	4.10–8.26	< 0.001
X6: HIV-related knowledge (0 = High,1 = Low)	1.57	1.35–1.83	< 0.001	1.28	1.08–1.51	0.004
X7: HIV-related stigma (0 = Low,1 = High)	1.94	1.66–2.28	< 0.001	1.50	1.26–1.77	< 0.001
X8: Testing history (0 = Never, 1 = Ever)	1.63	1.27–2.10	< 0.001	1.36	1.04–1.77	0.026
X9: Self-efficacy of condom use (0 = Low,1 = High)	0.80	0.68–0.94	0.008	0.70	0.59–0.84	< 0.001
X10: Monthly expenditure (0 = Low,1 = High)	2.85	2.34–3.46	< 0.001	2.55	2.07–3.14	< 0.001
X11: Residential areas (0 = Rural, 1 = Urban)	1.58	1.36–1.84	< 0.001	1.35	1.15–1.59	< 0.001
X12: Knowledge of local AIDS organization (0 = No,1 = Yes)	1.32	1.14–1.53	< 0.001	0.71	0.60–0.83	< 0.001
X13: Awareness of the national AIDS policy (0 = No,1 = Yes)	1.39	1.20–1.62	< 0.001	1.40	1.18–1.66	< 0.001

Those with more enabling resources [i.e., high levels of condom use self-efficacy (AOR = 0.70, 95% CI:0.59–0.84) and being knowledgable of local AIDS service organization (AOR = 0.71, 95% CI:0.60–0.83)] were less likely to report RD. However, out of our expectation, spending more than 2000 Yuan a month on basic needs (i.e., having higher expenditure)(AOR = 2.55, 95% CI:2.07–3.14), residing in urban areas (AOR = 1.35, 95% CI:1.15 -1.59) and being knowledgeable of the national AIDS policy (AOR = 1.40,95% CI:1.18–1.66) were associated with increased the discordance between perceived risk and risky sexual behaviors among undergraduates (Table 4).

## Discussion

### Main findings of this study

In this cross-selectional study, the overall discordance rate (8.5%) between perceived risk and actual risky sexual behaviors among college students in mainland China, which was a little higher than the rate (5.9%) calculated based on HIV- negative individuals in serodiscordant partnerships with clients attending an urban clinic in Uganda [7], but far lower than the level reported among high-risk population such as men who have sex with men (MSM) [1, 2, 8] and transgender women (81.4%) in Thailand [1]. Supposing that actually engaging in risky sexual behaviors [6, 7] was considered the gold standard for for diagnosing HIV acquisition risk, our findings suggested that self-perception of risk had relatively high negative predictive value (91.5%), possibly due to the fact that our survey was conducted in a low prevalence population (i.e., only 8.9% out of 10,665 eligible undergraduates were objectively scored as being at high risk [18]). Recognizing the existence of risk discordance and in an effort to reduce the number of undiagnosed HIV infections, public health professionals, community-based service providers and clinicians should rely upon both perceived and actual risk when assessing their patients' need for a HIV test [3]. Furthermore, an opt-out approach is strongly recommended to broaden the scope of HIV diagnostic and screening [23, 24].

Based on the Anderson's behavioral model, this study assessed the effects of eight predisposing characteristics (including gender, sexual orientation, major, grade, age at first sex, HIV-related knowledge and stigma, testing history) and five enabling variables (i.e., condom use self-efficacy, monthly expenditure, residence, knowledge of local AIDS organization, awareness of the national AIDS policy) on risk discordance among undergraduates in mainland China. Some of these findings are consistent with previous research.

Non-heterosexuals men were found to be more likely to exhibit risk discordance. The possible explanation for this finding may be the fact men who have sex with men (MSM), transgender women, and bisexual men have been listed as the target population for HIV prevention campaigns. However, HIV-related stigma is still highly prevalent among college students, as indicated by our previous study [18]. Therefore, low perceived risk might be merely an illusion created by non-heterosexuals men, totally out of touch with the reality of their actual risk-taking behaviors [2].

Beyond our expectation, major was found to have no statistically significant influence on risk discordance. One possible explanation for this phenomenon is that the current dominant biomedical model of health places greater emphasis on providing medical students with HIV/AIDS-related knowledge such as prevalence and incidence rates, modes of transmission, risk and protective factors [25], while topics regarding risk estimation, risk perception and adoption of safe sexual behaviors (e.g., taking PrEP and PEP to prevent new infections) are often ignored. As indicated in Table 2, major as a predispoing factor had statistically significant associations with a range of enabling variables such as condom use self-efficacy, monthly expenditure, knowledge of local AIDS organization and awareness of the national AIDS policy. Therefore, it can be inferred that major exerted indirect effects on risk discordance through these enabling variables (Fig. 1 and Table 2).

Consistent with the previous study, being older [13] and having the first sex at an earlier age [1, 8] was associated with increased risk discordance. This greater discordance could be explained by the increase of optimism bias in perceiving HIV risk. More specifically, older undergraduates and early initiators were exposed to sexual activity for a longer period and were also more likely than their respective counterparts to engage in risky sexual behaviors [14]. However, with the wide application of highly active anti-retroviral therapy, HIV has evolved from a fatal disease to a manageable chronic condition [26] and thus individuals may be more prone to engaging in risky sexual behaviors. Furthermore, a recent longitudinal study conducted by Levy et al. [27] has indicated that older age was associated with greater treatment optimism and also established an association between treatment optimism and subsequent sexual risk behaviors.

Higher levels of knowledge about HIV transmission, more positive attitudes and higher condom use self-efficacy were found to be associated with decreased risk discordance among undergraduates. This finding is not surprising as it fits the Anderson's behavioral model. Those with higher levels of knowledge and more positive attitudes towards PLHIV often gain a better understanding of the modes of HIV infection and prevention measures, know more about the skills of negotiating condom use and local AIDS organization and use this information to more effectively avoid risky sex and accurately evaluate the potential risk of HIV infection.

Contrary to previous studies [1, 5], having ever been tested for HIV was associated with increased risk discordance among undergraduates. This finding can be explained by the fact that screening for HIV is based upon their actual risk-taking behaviors, while negative rapid HIV test results, especially false-negative self-test results which were not often used in conjunction with post-test counseling, linkage to care and risk-reduction interventions [28], might have falsely reassured them that they were at lower risk of acquiring HIV than their actual behaviors indicate [5].

In order to achieve universal access to HIV/AIDS prevention, treatment and care services, China introduced the "Four Frees and One Care" AIDS policy [16]. In this study, being knowledgeable about the national AIDS policy was also associated with greater discordance, possibly due to decreased fear, shame and stigma surrounding HIV and AIDS [18], along with increased HIV treatment optimism [29–31]. Similarly, undergraduates with higher expenditure and those residing in urban areas were more likely to exhibit RD, because higher social economic status is characterised by better access to education, information and health services, which would produce cognitive bias such as optimistic bias, control illusion and overconfidence [1].

### Limitations

Several limitations need to be taken into consideration when interpreting and evaluating the results. First, since this study relied on a cross-sectional design to measure RD and its influencing factors simultaneously, it was difficult to infer a cause and effect relationship observed between these variables. Second, despite effort to obtain a large, geographically diverse sample across the whole country, undergraduates in this study were recruited using a combination of convenience and snowball sampling techniques and the majority of the respondents were from Hubei province, thus limiting generalization of the results. Third, due to the sensitivity of this topic, respondents might underreport risky sexual behaviors, thus contributing to an underestimation of the extent and severity of discordance between self-perceived and actual risk of HIV infection. Fourth, individuals having engaged in at least one type of risky sexual behaviors were classified into the RD group. Thus, the discordance rate between perceived risk and actual sexual risk behaviors might be exaggerated. Fifth, besides consistent condom use, some high-risk individuals such as men who have sex with men (MSM) might choose to take pre-exposure prophylaxis (PrEP) and post-exposure prophylaxis (PEP), which were not assessed in this study. Hence, this might inflate the RD group. Nonetheless, PrEP and PEP were not widespread and well known in mainland China during the time of the study [32]. Sixth, psychosocial factors (e.g., denial, dissociation and optimistic bias), HIV status and sexual behaviours of each partner were not obtained in this study and therefore could not be assessed as possible explanations for risk discordance. Seventh, participants with high perceived risk were excluded from this analysis, because a different approach is required to facilitate their health-seeking behaviour when compared to those with low perceived risk [1] and also due to insufficient sample size. Eighth, 1595 participants who failed to assess their risk level were exclude from this analysis, which might result in a substantial decrease in the sample size and biased estimation of the discordance rate and the odds ratios. However, when including those failing to assess their risk level as having low perceived risk [14] and running the same logistic regression model, the discordance rates between 8808 undergraduates included in the present analysis and those (1595) excluded because they did not assess their risk level were similar (8.5% vs. 8.8%, *P* = 0.692), and all of the independent variables did not change the direction of their associations, but altered their strengths slightly. Therefore, there was no evidence of significant selection bias due to the exclusion. Ninth, HIV testing history was measured, but the testing results for previous testers were not verified. Finally, perception of HIV risk is not static, but varies with context as well as over time. Even in the same level of actual risk, individuals differ in their perceptions [14].

### Implications of the study

In spite of the above-mentioned limitations, the findings from our study have several implications for the design and implementation of HIV risk reduction programs on college campuses. To the best of our knowledge, ours is the first to determine the extent of discordance between perceived and actual HIV risk and the factors associated with this discordance among a large undergraduate sample selected from 30 provinces throughout the whole country. The findings suggested that non- heterosexual men, non-freshmen, early initiators of sexual intercourse, and those who had lower levels of HIV knowledge, displayed higher levels of stigma against PLHIV and had ever been tested for HIV were more prone to reporting RD. Those with more enabling resources (i.e., high levels of condom use self-efficacy and being knowledgable of local AIDS organization) were less likely to report RD. However, spending more than 2000 Yuan a month on basic needs, residing in urban areas and being knowledgeable of the national AIDS policy increased the chance of risk discordance. Therefore, four types of intervention measures are recommended to reduce the discordance between perceived and actual HIV risk and finally to reach the goal of Zero AIDS.

- (1) Make opt-out HIV testing routine in college campus: the HIV testing and counseling should be offered routinely to all undergraduates unless they decline (i.e., opt-out screening) [23, 24], because it not only diagnoses those who may under- estimate their HIV risks, but also helps to destigmatize HIV testing [33]. It was also noted by Golin and his colleagues [5] that post-test counseling has not become an dispensable part of the opt-out testing strategy recommended by Centers for Disease Control and Prevention. Therefore, assessment of the discordance between perceived and actual HIV risk and information regarding continued risk of HIV acquisition despite a negative result should be incorporated into opt-out testing strategy [5].
- (2) Target students with high-risk characteristics and improve the acceptability of PrEP and PEP: Non-heterosexual men, non-freshmen, early sexual initiators and those with higher expenditure and residing in urban areas had a higher tendency to exhibit RD and were classified as high-risk individuals and should therefore be prioritized for PrEP and PEP [34]. It was reported by Yang (2019) that PrEP and PEP have not been widely accepted and implemented among the target population in China [32], despite being generally safe and efficient in preventing new infections. In order to improve their acceptability and utilization among high-risk individuals, other issues such as high costs and low levels of awareness must be adequately addressed [35, 36].
- (3) Enhance condom use self-efficacy: the simplicity and effectiveness of consistent condom use makes it become one of the comprehensive and sustainable approaches to the prevention of HIV and other sexually transmitted diseases. In order to promote consistent use of condoms, the first and foremost thing is to teach communication skills to undergraduates. Also, the intervention should focus on their perceived barriers to communicating to sexual partners about condom use and developing strategies appropriate to make them feel at ease with the negotiating process and thus enhancing condom use self-efficacy [37].
- (4) Conduct health education: health education program [38] appears to be useful in raising their knowledge about HIV/AIDS, reducing stigma towards PLHIV, and also increasing the awareness of the benefits of early diagnosis, the advancements in antiretroviral treatment, and the national AIDS policy that guarantees universal access to prevention, treatment, care and support.

## Conclusions

Drawing on a large, geographically diverse sample of college students from across China, this study suggested that the overall discordance rate was 8.5%. Compared with non-heterosexual men, non-heterosexual women, heterosexual men and women were less likely to exhibit RD. Furthermore, higher levels of knowledge, lower levels of stigma towards PLHIV and higher condom use self-efficacy decreased the odds of exhibiting RD. However, spending more than 2000 Yuan one month on basic needs, residing in urban areas, being non-freshmen, being less than 14 years old at sexual debut, having ever been tested for HIV and being knowledgeable of the national AIDS policy increased the chance of reporting RD. Comprehensive interventions, including targeting students with high-risk characteristics, improving the acceptability of PrEP and PEP, conducting health education, enhancing self-efficacy for using condoms and making opt-out HIV testing routine in college campus, should be taken to reduce the discordance between perceived and actual HIV risk and finally to reach the goal of Zero AIDS.

## Data Availability

The data set supporting the results of this article is available in the Harvard Dataverse repository at: https://dataverse.harvard.edu/dataset.xhtml?persistentId=doi:10.7910/DVN/QKD452

## Abbreviations

RD: Risk discordance
AOR: Adjusted odds Ratio
COR: Crude odds ratio
CI: Confidence Interval
RD: Risk discordance
HIV: Human Immunodeficiency Virus
KABP: Knowledge-attitude-belief-practice
HUST: Hubei University of Science and Technology
MSM: Men who have sex with men
PLHIV: People living with HIV
PrEP: Pre-exposure prophylaxis
